# A Core Flood and Microfluidics Investigation of Nanocellulose as a Chemical Additive to Water Flooding for EOR

**DOI:** 10.3390/nano10071296

**Published:** 2020-07-01

**Authors:** Reidun C. Aadland, Salem Akarri, Ellinor B. Heggset, Kristin Syverud, Ole Torsæter

**Affiliations:** 1 PoreLab Center of Excellence, Department of Geoscience and Petroleum, Norwegian University of Science and Technology (NTNU), N-7491 Trondheim, Norway; salem.s.f.akarri@ntnu.no (S.A.); ole.torsater@ntnu.no (O.T.); 2 RISE PFI, N-7491 Trondheim, Norway; ellinor.heggset@rise-pfi.no (E.B.H.); kristin.syverud@rise-pfi.no (K.S.); 3 Department of Chemical Engineering, Norwegian University of Science and Technology (NTNU), N-7491 Trondheim, Norway

**Keywords:** enhanced oil recovery, chemical flooding, nanocellulose, cellulose nanocrystals, TEMPO-oxidized cellulose nanofibrils, microfluidics

## Abstract

Cellulose nanocrystals (CNCs) and 2,2,6,6-tetramethylpiperidine-1-oxyl (TEMPO)-oxidized cellulose nanofibrils (T-CNFs) were tested as enhanced oil recovery (EOR) agents through core floods and microfluidic experiments. Both particles were mixed with low salinity water (LSW). The core floods were grouped into three parts based on the research objectives. In Part 1, secondary core flood using CNCs was compared to regular water flooding at fixed conditions, by reusing the same core plug to maintain the same pore structure. CNCs produced 5.8% of original oil in place (OOIP) more oil than LSW. For Part 2, the effect of injection scheme, temperature, and rock wettability was investigated using CNCs. The same trend was observed for the secondary floods, with CNCs performing better than their parallel experiment using LSW. Furthermore, the particles seemed to perform better under mixed-wet conditions. Additional oil (2.9–15.7% of OOIP) was produced when CNCs were injected as a tertiary EOR agent, with more incremental oil produced at high temperature. In the final part, the effect of particle type was studied. T-CNFs produced significantly more oil compared to CNCs. However, the injection of T-CNF particles resulted in a steep increase in pressure, which never stabilized. Furthermore, a filter cake was observed at the core face after the experiment was completed. Microfluidic experiments showed that both T-CNF and CNC nanofluids led to a better sweep efficiency compared to low salinity water flooding. T-CNF particles showed the ability to enhance the oil recovery by breaking up events and reducing the trapping efficiency of the porous medium. A higher flow rate resulted in lower oil recovery factors and higher remaining oil connectivity. Contact angle and interfacial tension measurements were conducted to understand the oil recovery mechanisms. CNCs altered the interfacial tension the most, while T-CNFs had the largest effect on the contact angle. However, the changes were not significant enough for them to be considered primary EOR mechanisms.

## 1. Introduction

The majority of existing oil fields are in their tail-end production, where most of the easily accessible oil has already been produced. The remaining oil is difficult to recover, and there is a low proportion of new fields for exploration, as most of the basins that might contain oil have already been explored, and many of the unexplored basins lie in remote and environmentally sensitive areas of the world (e.g., the Arctic) [1]. Therefore, it is important to try to extend the lifetime of already operating fields. For example, the average recovery rate for the oil fields on the Norwegian continental shelf is approximately 50%, thus, resulting in a high amount of unrecovered oil that is not producible with the current technology [2]. The application of enhanced oil recovery (EOR) techniques entails methods that could improve the oil recovery. Research and technological development have been directed to advance the techniques of enhanced oil recovery. Recently, nanocelluloses have been introduced as environmentally friendly nanoparticles for EOR applications [3,4,5,6,7].

Cellulose is an abundant biopolymer derived from various sources, usually from wood. A tree produces 13–14 g of cellulose per day, and the total production of cellulose all over the world is estimated to be 7.5 × 10^10^ tons per year [8,9]. In the cell wall of plants, cellulose molecules are formed as solid structures made from bundles of cellulose molecules held together by inter- and intramolecular hydrogen bonds, with dimensions in the nanoscale. Today, it is possible to extract these nanoscaled structures from plants by various methods. Depending on the production strategy, different structures of nanocellulose can be formed. By subjecting cellulose to controlled acid hydrolysis, cellulose nanocrystals (CNCs) can be created, while cellulose nanofibrils (CNFs) can be obtained by using high shear forces, e.g., a high-pressure homogenizer [10], often after a chemical pretreatment has been applied. CNCs from wood are usually 3–5 nm wide, and have lengths ranging from 100–200 nm [11], while CNFs usually have a diameter in the range of 5–60 nm and lengths of several micrometers [10]. The OSPAR Commission reported that CNCs and CNFs pose little or no risk to the offshore environment [12].

Thermal stability is an important attribute when testing new particles for EOR purposes, as temperatures in reservoirs can get quite high (>90 °C) [13]. Heggset et al. [14] studied the temperature stability of CNCs and CNFs, where it was found that both types of particles exhibited superior temperature stability when compared to e.g., the biopolymer xanthan. Furthermore, in another study using modified nanocellulose, it was found that when nanocellulose was subjected to elevated temperatures it experienced a slower loss in viscosity compared to the synthetic polymer, hydrolyzed polyacrylamide (HPAM) [15]. Therefore, CNCs and CNFs might serve as a sustainable and effective alternative EOR technique for offshore oil fields.

Molnes et al. [6] did core-flood experiments using CNCs and observed a slight increase in oil recovery (3.4%) when the nanofluid was injected as a tertiary recovery agent. Aadland et al. [3] did a high-temperature core flood with CNCs and showed that the nanoparticles contributed to a slight increase in incremental oil recovery (1.2%). Kusanagi et al. [5] did tertiary core flood experiments using 2,2,6,6-tetramethylpiperidine-1-ox (TEMPO)-oxidized cellulose nanofibers (T-CNFs) and found that additional oil was produced (8.6%). However, they observed filtration in the porous media and poor injectivity. Experiments using surface-functionalized nanocellulose showed that the particles contributed to a 3–17% increase in oil recovery when injected after water flooding. From microfluidic experiments using the same particles, three main oil recovery mechanisms were discovered: emulsification, dragging, and wettability alteration [16]. Emulsification was also one of the main findings from a previous study Wei et al. [15] conducted.

A recovery agent, i.e., nanofluid or low salinity brine, can be injected in a secondary or tertiary mode. Secondary mode corresponds to the injection at initial water saturation and mainly displaces a large connected body of oil in the porous medium, while the tertiary mode is the injection after reaching residual oil saturation via the secondary-mode fluid, aiming to mobilize the remaining trapped oil clusters. Therefore, the response to injecting a recovery agent differs according to the mode, as shown in several studies [17,18,19,20,21,22]. In addition, the response is also significantly controlled by the wettability condition of the rock, as it affects the initial distribution of fluids within the porous medium, as well as the displacement dynamics [23,24,25,26,27,28]. Several studies tested recovery agents at different wettability conditions, i.e., water wet and intermediate wet, however, the reported results were not consistent [19,20,21,29,30]. A study suggested that the effect of aging, in terms of oil recovery, was dependent on the oil-brine-rock system, where, for example, rocks of the same type with different clay content would show different responses. As previously mentioned, the temperature is another important parameter in relation to oil recovery, which also affects the recovery response. Therefore, it is vital to establish an understanding of how new EOR candidates behave in different recovery modes, wettability conditions, and temperatures.

Mobilization of the residual oil in a porous medium is considerably governed by the capillary number (N_C_) [31]. It is defined in Equation (1) [32]; where *q_w_* is the interstitial velocity (m/s), *µ_w_* is the viscosity (Pa s) and *σ_ow_* is the interfacial tension (N/m) between oil and water. The flow in a porous medium is dominated by viscous forces if the capillary number is high, and capillary forces if the capillary number is low. A higher capillary number can be achieved by decreasing the interfacial tension, or by increasing the viscosity or the velocity of the displacing phase. The capillary number at the end of a water flood usually ranges from 10^−6^ to 10^−4^ [33].

(1) $$N_{C}=\frac{q_{w}\times \mu_{w}}{\sigma_{ow}}$$

The capillary number is directly related to the amount of trapped oil in a porous medium.

For brine flooding, studies [34,35] have shown that high capillary numbers (achieved by increasing the injection rate) induced lower volumes of trapped oil in natural rocks and glass-bead packs. However, for nanofluids a different trend was observed, where high capillary numbers yielded lower oil recovery factors. The low oil recovery was explained by that nanoparticles require sufficient time for altering wettability via structural-disjoining pressure, or that they agglomerate at high injection rates [35,36].

Microfluidic micromodels have been a vital technique in EOR applications, since they provide micro-visualization of the fluid flow behavior, which can be recorded for qualitative observation, quantitative analysis, and simulation studies. Microfluidic micromodels have been used for assessing surfactant-polymer flooding mixed with nanoclay for improving heavy oil recovery [37], evaluating polymer EOR for unconsolidated sand reservoirs [38], investigating EOR mechanisms associated with injecting silica nanoparticles [39], and the screening of surface-modified silica nanoparticles for EOR [40]. They also have been used to study the effect of the polymer concentration and injection rate on the sweep efficiency [41], examining the impact of fluid rheology on oil recovery [42], and simulation of fluid configurations captured from imbibition and drainage experiments on a micromodel [43].

In addition to the recovery factor, the acquired images from microfluidic studies can be processed to evaluate the cluster size distribution and connectivity of the non-wetting phase. Cluster size distribution is affected by wettability condition [44], injection rate [34], and interfacial tension [45]. The connectivity of the non-wetting phase in a porous medium can be described by the Euler characteristic/number (E) [46,47]. For 2D images, the Euler number is given by Equation (2), where C is the number of isolated components in the image, and H is the number of holes within the components [47,48]. The aforementioned factors affecting the cluster size distribution might influence the connectivity, since Equation (2) is dependent on the number of clusters in the image.

E = C − H(2)

In the current experimental study, core floodings and microfluidic experiments have been conducted, to further investigate the potential of nanocelluloses for enhanced oil recovery. On the core-scale, the main objective was to perform a comprehensive study on CNCs as recovery agent in a secondary and tertiary mode, in water-wet and intermediate-wet systems, and at high and low temperatures. In addition, a novel approach was tested for one core plug, where the idea was to reuse the same natural pore structure (same core plug) after wettability restoration, excluding the pore architecture effect on trapping efficacy. Furthermore, T-CNFs, which are of different structure and size compared to CNCs, were evaluated on core-scale to see their ability in the mobilization of trapped oil, compared to CNCs at the same conditions. Moreover, 2D glass microchips of the same pore-structure and wettability were used to obtain micro-scale comparisons between the injection of brine, CNCs, and T-CNFs. The studied micro-scale parameters were dynamic changes in the oil connectivity, oil recovery, and residual cluster size distribution. The microchips were also used to show the effect of a higher flow rate on oil recovery, size distribution, and oil connectivity. This study aims to contribute towards filling the knowledge gap in the nanocellulose literature for EOR applications.

## 2. Materials

### 2.1. Rock

The core plugs used in this study were extracted from a Berea sandstone block and had an average length and diameter of 10 cm and 3.8 cm, respectively. The block was acquired from a quarry in Ohio, USA and was purchased from Berea Sandstone Petroleum Cores (Berea Sandstone Petroleum Cores, Vermilion, OH, USA). The core plugs were rinsed in a Soxhlet apparatus with methanol and dried in an oven at 60 °C, prior to the core floods. Permeability and porosity measurements were performed on the dry core plugs. Core properties are listed in Table 1.

### 2.2. Microfluidic Chip

Five borosilicate glass microfluidic chips of the same pore-network structure were used in this study (Micronit Microfluidics, Enschede The Netherlands). The dimensions of the chip are 45 × 15 × 1.8 mm. The chips contain a porous medium (20 × 10 × 0.02 mm) with a pore-network structure representing actual rock-pore structures. The pore volume, permeability, and porosity are 2.3 µL, 2.5 D, and 57%, respectively.

### 2.3. Brine

All experiments were performed using low salinity water (LSW), which consisted of 0.1 wt.% sodium chloride (NaCl) prepared from NaCl (Sigma-Aldrich, St. Louis, MO, USA) and de-ionized water (DIW). Properties are listed in Table 2.

### 2.4. Nanocelluloses

#### 2.4.1. Cellulose Nanocrystals

**Figure 1:**
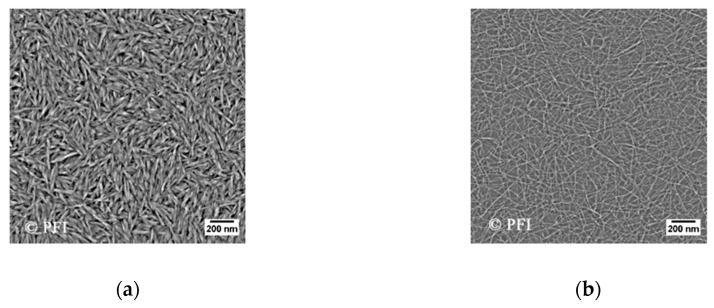
Atomic force microscopy (AFM) images of cellulose nanocrystals (**a**) and 2,2,6,6-tetramethylpiperidine-1-oxyl (TEMPO)-oxidized cellulose nanofibrils (**b**). From the images, it can be seen that both cellulose nanocrystals (CNCs) and TEMPO-oxidized cellulose nanofibrils (T-CNFs) consists of elongated particles, and that T-CNFs are longer compared to the CNCs. They both exhibit anisotropic properties. The procedure for how the AFM images were obtained is explained in Aadland [53].

The cellulose nanocrystals (CNCs) were acquired from the University of Maine. The material was manufactured at the Forest Products Laboratory in Madison, USDA (U.S. Dep. of Agriculture, USA). The CNCs were produced by acid hydrolysis of softwood pulp, where 64% (by mass) sulphuric acid was used to hydrolyze amorphous regions of the cellulose material, yielding acid-resistant crystals [49]. The stock-dispersion had a concentration of 12 wt.%. Properties are listed in Table 2 and Table 3, and an atomic force microscopy (AFM) image of the particles can be seen in Figure 1. In the experiments, the nanofluid was diluted to 1.0 wt.% CNCs using LSW.

#### 2.4.2. TEMPO-Oxidized Cellulose Nanofibrils

TEMPO-oxidized cellulose nanofibrils (T-CNFs) were produced at RISE PFI (Trondheim, Norway). For the production of T-CNFs, never-dried, bleached softwood pulp fibers were used as the source material. The preparation was performed using 2,2,6,6-tetramethylpiperidine-1-oxyl (TEMPO) radical-mediated oxidation, as previously described by Isogai et al. [50]. The TEMPO-oxidized pulp was afterwards pretreated in a Mazuko-grinder before further fibrillation. The fibrillation was done using a Rannie 15 type 12.56× homogenizer (APV, SPX Flow Technology, Silkeborg, Denmark), and the samples were fibrillated for four passes with a pressure drop of 1000 bar in each pass. Carboxylate content was determined using conductometric titration as previously described [51,52]. The equipment used was a 902 Titrando (Methrom AG, Herisau, Switzerland), an 856 conductivity module and Tiamo software (Metrohm AG, Herisau, Switzerland). The stock dispersion of T-CNFs had a concentration of 0.66 wt.% and was diluted to 0.1 wt.% using LSW. Properties are listed in Table 2 and Table 3, and an AFM image of the particles can be seen in Figure 1.

From the particle size measurement with dynamic light scattering (DLS), it was observed that CNCs are, to a greater extent, a monodisperse sample, while T-CNF was polydisperse. DLS is not considered a good technique to determine the size of these rod-shaped particles, as it is focused towards spherical particles. Nevertheless, it gives a relative particle size and allows us to compare the particles against each other. From the obtained AFM image (Figure 1b) it can be seen that the diameter for T-CNF is approx. 15 nm, which is within the range that has been reported before in the literature for cellulose nanofibrils. Cellulose nanofibrils usually have a diameter in the range of 5–60 nm, and can be several micrometers in length [10].

The obtained characteristics for the CNCs are in accordance with result from the previous literature [7,55,56].

### 2.5. Oil

Two types of crude oil were used during the experiments, denoted crude oil C and crude oil D. Both were taken from the same field in the Norwegian Sea, but they exhibit different properties (Table 3). The oils were filtered twice through a five µm Millipore filter under vacuum to remove impurities. A saturates, aromatics, resins, asphaltenes (SARA) analysis was performed for both the oils, and can be found in Table 4. From the analysis, it was seen that crude oil D contained much fewer resins and asphaltenes compared to crude oil C. Resins and asphaltenes are polar compounds, which are considered the surface-active components in crude oil [57].

## 3. Experimental Methods

### 3.1. Fluid Interaction Measurements

#### 3.1.1. Fluid-Fluid Interactions

Interfacial tension (IFT) was measured using the drop shape analyzer DSA 100S with acquisition software (Kruss GmbH, Hamburg, Germany). The IFT was automatically calculated in the software ADVANCE (Kruss GmbH, Hamburg, Germany), by applying the pendant drop technique, where the contour of the drop was determined with image analysis. Measurements were taken every five minutes for 12 h. The experiment was conducted at ambient temperature and pressure.

#### 3.1.2. Fluid-Solid Interactions

The contact angle was measured using the same apparatus and acquisition software that was applied for IFT. The measurement cell was filled with the respective fluid (i.e., brine or nanocellulose) and an oil droplet was placed underneath a glass substrate. The static contact angle was measured using the captive bubble method with the software ADVANCE. The contact angle was measured at 5 min intervals for 12 h and was performed at ambient conditions.

### 3.2. Core Flood Study

#### 3.2.1. Experimental Setup

**Figure 2:**
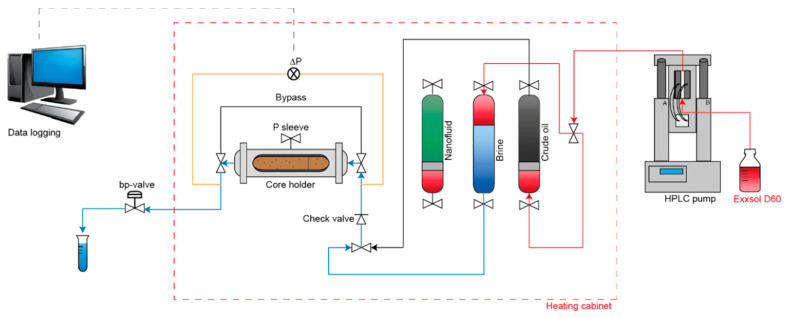
Schematic illustration of the experimental setup of the core flood.

The experimental setup of the core floods can be seen in Figure 2. A high-performance liquid chromatography (HPLC) pump (Teledyne ISCO, Lincoln, NE, USA) was used, with Exxsol™ D60 (ExxonMobil Chemical Europe, Machelen, Belgium) as pump fluid. Three cylinders were installed inside the heating cabinet, containing the relevant fluids-crude oil, brine, and nanofluid, respectively. For crude oil and nanofluid, a piston was installed inside the cylinder to prevent mixing with Exxsol™ D60. Prior to each flooding stage, the respective fluid was flooded through the bypass line and out to the effluent collector to reduce the dead volume. The back-pressure valve was only installed and applied during the high-temperature floods.

The core was installed horizontally in a Hassler core cell and sleeve a pressure within 20–25 bar was maintained. The pressure was logged across the core throughout the experiment, and effluent samples were collected every one-fourth pore volume (PV) during water- and nano flooding.

#### 3.2.2. Core Flood Experiments

Ten core flood experiments were conducted, where the overall goal was to determine if nanocelluloses have potential as enhanced oil recovery agents. The experiments were designed to test the performance of the fluid under various conditions. The injection mode, type of oil, temperature, wettability of rock, and particle type were the variables investigated. The experiments were classified into three parts based upon their research objectives (see Table 5 for details).

The first part compares the induced oil recovery by CNCs to brine at fixed conditions, including the pore structure via reusing the same core plug. The second part aims at exploring the response of CNCs as a recovery agent in a secondary and tertiary mode, in water-wet and intermediate-wet systems, and at high and low temperatures. The third part compares T-CNFs, to CNCs in terms of the ability to mobilize trapped oil under the same conditions.

#### 3.2.3. Core Flood Procedure

A 100% brine saturated core was installed in the core holder. Initial saturations were established by drainage using crude oil injected at two different flow rates (1.0 and 10 mL/min). The oil recovery experiment was conducted either as a secondary-or tertiary recovery technique, where the respective fluid was injected using two flow rates (0.3 and 3.0 mL/min). Each flow rate continued until pressure stabilized and no more oil was produced. For secondary mode recovery, LSW or nanofluid was injected after establishing initial conditions in the core. Tertiary mode recovery consisted of injecting LSW as secondary mode, then injecting the nanofluid. After each nanoflood stage (secondary or tertiary) a post flush with LSW was injected at 3.0 mL/min, to see if any incremental oil could be produced as a result of creating a new flow pattern in the core induced by the nanoparticles.

#### 3.2.4. Aging

Four cores were aged using crude oil D (Test 4, 5, 8, and 9). Aging was done to make the cores more intermediate wet. First, initial saturations were established in the core using the same procedure as explained in the previous section. Afterwards, the core was placed in a container and immersed in crude oil D. The container was stored in an oven at 60 °C. Three of the cores were aged for five weeks (35 days), while one core was aged for seven weeks (49 days).

### 3.3. Microfluidic Study

#### 3.3.1. Experimental Setup

**Figure 3:**
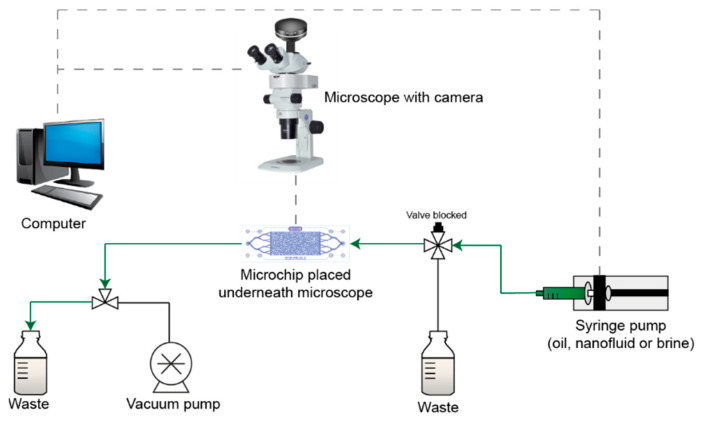
Schematic illustration of the experimental setup of the microfluidic experiment.

The experimental setup of the microfluidic experiment is presented in Figure 3. A Micronit EOR platform was used to conduct the microfluidic experiments under a microscope (Olympus SZX7, Olympus, Langhus, Norway) integrated with a digital camera (Olympus UC90, Olympus, Langhus, Norway). Olympus Stream Basic 2.1 (Olympus, Langhus, Norway) was used for image acquisition, as well as experiment monitoring, via a live feed. A syringe pump (Harvard Apparatus Pump 33 DDS, Holliston, MA., USA) and CODAN luer-lock syringes were used for the fluid injection. The syringe was connected to a 4-way valve, which was used to bleed the line from the syringe when changing to a new fluid. The production line was connected to a three-way valve directing the fluids to a waste beaker during the experiment or allowing the vacuuming of the system using Edwards RV3 vacuum pump (Edwards, Lørenskog, Norway).

#### 3.3.2. Microfluidics Experiments

Five microfluid experiments were conducted at room temperature, which are classified into two groups: high injection-rate and low-injection rate experiments. They were designed to evaluate the dynamic change in oil recovery, connectivity, and clustering, enabling a comparison between the three injected fluids during the low rate injection scheme. In addition, it aims at evaluating the performance of the nanofluids when they are injected with a high rate. Table 6 presents the details of the injection and image acquisition. The analyzed area, which is with the same pore structure for the five experiments, was selected in the middle of the microchip to avoid capillary end effects.

#### 3.3.3. Microfluidic Procedure

After inserting a clean and dry microfluidic chip in the flooding platform, the system was vacuumed to remove the air through the production line. After that, brine (0.1 wt.% NaCl) was injected to fully saturate the chip at a constant rate of 0.1 mL/min. It was followed by injecting the non-wetting phase at three different flow rates (0.006, 0.06, and 0.5 mL/min) to establish initial oil and irreducible water saturations in the microchip. After acquiring an image of the initial state, a secondary recovery was performed. During the recovery stage, images were taken to capture the dynamic changes.

#### 3.3.4. Image Processing and Analysis

**Figure 4:**
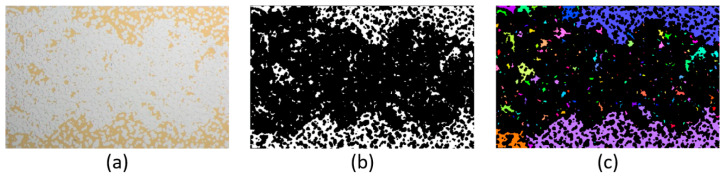
(**a**) Pre-processing image of a microchip (oil in gold, glass & brine in white), (**b**) a post-segmentation binary image (oil in white), and (**c**) oil clusters were labeled by pseudorandom colors for qualitative analysis. Quantitative analysis was applied to the binary image (**b**).

The acquired images were processed and analyzed using Fiji [58] with an installation of BoneJ [59] plugin. The images were firstly segmented using a color thresholding method to extract the non-wetting phase. The resulted binary images were utilized for cluster analysis, in order to calculate the total area of the oil (*A*_o_), cluster size distribution, number of clusters (C), number of holes (H), and size of clusters. Figure 4 illustrates an image of a saturated microchip through the process. Based on these parameters, the change in oil recovery, oil connectivity, and cluster number were evaluated as a function of pore volume injected (PVI). The oil recovery is given by Equation (3). The oil connectivity is described by the Euler number, see Equation (2). In this work, the Euler number is normalized by the Euler number of the oil at the initial stage i.e., pre-imbibition. A normalized Euler number (X) of 1 represents the pre-flooding stage, which is the maximum value and state of connectivity, and the more disconnected the oil becomes, the farther the value is from 1. For example, the non-wetting phase with a connectivity of X = 0.2 is more disconnected than X = 0.4. In addition, the normalized cumulative residual oil volume as a function of oil cluster size was obtained.

(3) $$Oil\;recovery=\frac{A_{o}{}_{PVI=0}-A_{o}{}_{PVI\geq 0}}{A_{o}{}_{PVI=0}}$$

## 4. Results and Discussion

### 4.1. Fluid-Fluid Interactions

Interfacial tension of LSW-crude oil system was 19.2 (±0.02) and 15.3 (±0.03) mN/m for crude oil C and D, respectively. These are considered reference values for the specific oil type, to which the nanoparticles were compared. Crude oil D had fewer asphaltenes compared to crude oil C, and overall lower IFT values were obtained with crude oil D. This trend in IFT based on oil composition has also been reported in the literature [60].

The same tendency in IFT was seen for both oil types (Table 7), with 0.1 wt.% T-CNFs being similar to the reference value and 1.0 wt.% CNCs being lower. A low IFT is favorable, as it will increase the capillary number, which, in turn, will help to mobilize more oil. However, the decrease in IFT using CNCs was not of orders of magnitude, which is a necessary requirement for it to be considered a primary recovery mechanism [61]. Nevertheless, some studies have also shown that an ultralow IFT might not be necessary to improve the oil recovery [62].

### 4.2. Fluid-Solid Interactions

The contact angle is considered to be the most universal measurement of the wettability of a surface and it is an approach to measure reservoir wettability [26]. Wettability alteration has often been ascribed to the asphaltene content in crude oil, where crude oil with a high asphaltene content renders often more oil-wet surfaces [57]. However, this was not observed in the current study, as crude oil C yielded lower contact angle values compared to crude oil D (Table 7).

From Table 7 it is seen that the contact angle for LSW-crude oil-glass system, was 49.6° (±0.2°) and 52.5° (±0.8°) for crude oil C and D, respectively. These are considered reference values. Looking at crude oil C, all final values (12th hour) were similar to the reference value. For crude oil D, the addition of nanoparticles to the solution caused a small increase in the contact angle, with T-CNFs having the highest value. A higher value in the contact angle is equivalent to the system becoming less water-wet. However, in this case, the system was still in the water-wet regime.

### 4.3. Capillary Number

The capillary number for the core floods can be found in Table 8. A normal water flood has a capillary number that is in the order of 10^−7^ [63]. To mobilize the remaining oil, the capillary number should be in the range of 10^−5^ or higher [61]. For the low rate water and nano floods, the capillary number was 10^−6^, while it was increased to 10^−5^ for the high rate floods. Thus, the increase in rate is expected to result in higher incremental oil production.

Looking at the effect of particle type and crude oil type, the capillary number was slightly higher for T-CNFs, compared to CNCs. In addition, crude oil D had slightly higher numbers than crude oil C, but they were all in the same order of magnitude.

### 4.4. Core Flood

#### 4.4.1. Part 1

The objective of the secondary recovery floods was to see if CNC nanofluid produced more oil compared to low salinity water.

The trapping efficiency of a porous medium is significantly associated with the structure of the pore-space through factors such as pore body-throat aspect ratio and pore coordination number [64,65,66,67]. Therefore, the decision was taken to reutilize Core 1 for testing the 1 wt.% CNC nanofluid, via rinsing the core with methanol and toluene using a Soxhlet apparatus, and then drying the core at 60 °C. After drying, a new permeability and porosity measurement were obtained to compare with the original measurements (Table 9). The wettability restoration method applied to the core was considered successful, since it led to the same irreducible water saturation after primary drainage (Table 10). Although Core 1 permeability and porosity was reduced by 6.5% and 1.1%, respectively, it is assumed that the pore structure and wettability effect on oil recovery is minimized by using this approach.

**Figure 5:**
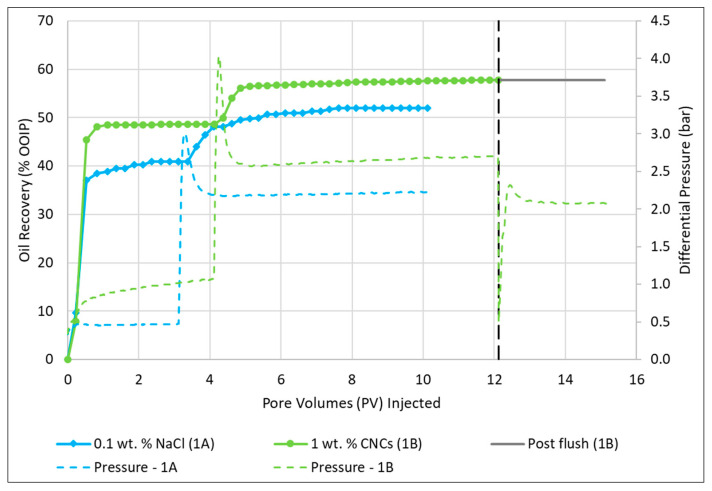
Oil recovery and pressure curves for secondary water flood (Test 1A) and secondary CNC nano flood (Test 1B). The black dotted vertical line indicates where post water flush was initiated for the nano flood. The spike in pressure curves indicates where the rate was switched from 0.3 mL/min to 3.0 mL/min, for the respective floods.

By looking at the pressure curves for secondary water- and CNC nano flood (Figure 5), the pressure was higher for the CNC nano flood stage, and it was slightly increasing for both the low rate and the high rate. However, no more oil was produced even though the pressure increased. Looking at the recovery, water flooding resulted in 52.0% total recovery, where 40.9% oil was produced during low rate and 11.1% oil was produced during the high rate (Table 10). For the second test using 1.0 wt.% CNC nanofluid (Test 1B), a higher recovery during the low rate (48.6%) and similar recovery to water flooding during the high rate (9.2%) were observed, overall resulting in a slightly higher total recovery of 57.8% oil (Table 10).

With this method, the nanofluid yielded 5.8% of original oil in place (OOIP)—more oil compared to low salinity water (Table 10). The increased oil recovery could either be a result of the viscosity difference between water and nanocellulose, or it could be a result of a physical interaction between the stiff and solid nanocellulose particles and the oil droplets. Another explanation for the increased oil recovery may be that pore throats could be blocked by the nanocellulose particles, leading the fluid in other directions.

This approach was only performed on one core as it was a time-consuming process and uncertainties regarding the wettability of the core arose. Soxhlet extraction can sometimes change the core from a water-wet state to an oil-wet state. In addition, the solvent may not contact all of the core [68].

#### 4.4.2. Part 2

In Part 2, CNC particles were used, and the parameters that were varied were wettability of the rock, temperature, and injection scheme. The results from this part are presented according to the injection mode.

##### Secondary Recovery

**Figure 6:**
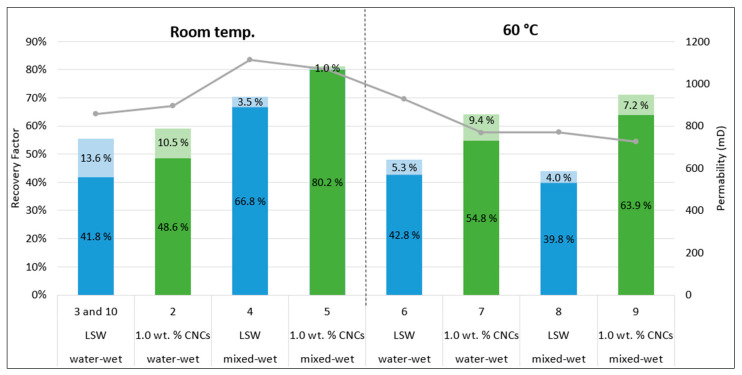
Recovery factors (% of original oil in place (OOIP)) and permeability of secondary recovery experiments. The vertical black dotted line acts as a marker between the experiments conducted at room temperature and 60 °C. Dark blue or dark green colors represent the recovery factor achieved from the low rate flooding (0.3 mL/min), while the lighter colors denote the recovery from the high rate flooding (3.0 mL/min). The numbers on the *x*-axis corresponds to the test number.

Further investigations of secondary recovery floodings were commenced by varying the wettability of the rock and temperature. In Figure 6, the recovery factors and permeability of the conducted experiments are illustrated. It is observed that CNC nanofluid produced more oil than LSW, when compared with the equal parallel experiment (Figure 6 and Table 10).

Four cores were aged in total, to make them more mixed-wet. Three cores were aged for five weeks (35 days); Test 4, 5, and 8, while Test 9 was aged for seven weeks (49 days). From an USBM wettability experiment Anderson [57] conducted on a Berea sandstone core, the wettability changed from water-wet (W = 0.8) to moderately oil wet (W = −0.3) over a 40-day period. However, after 35 days, the value was approximately −0.15, which indicates that the core was neutrally to moderately oil-wet. Thus, the chosen aging time in the current experimental study should be sufficiently long to alter the wettability to some degree. However, since it is uncertain if the wetting equilibrium has been reached, these cores will be considered intermediate or mixed-wet.

By looking at the effect of oil recovery and rock wettability, it seemed like the nanofluid was able to produce more oil when the core had been aged, and this was the case for both the room temperature and 60 °C flood. For LSW, on the other hand, no clear trend was observed, as the experiments at room temperature yielded a higher oil recovery for the aged core (Test 4), while, at 60 °C, more oil was produced with the water-wet core (Test 6). However, it should be noted that the permeability difference between the water-wet core and the mixed-wet core at 60 °C using LSW was approximately 150 mD. In addition, the total oil recovery was similar to each other—48.1% (Test 6) versus 43.8% (Test 8). A higher permeability for core 8 could, therefore, have resulted in a slightly higher recovery, which, in turn, could have given the same trend as was observed with the nanofluid.

All the room temperature floods resulted in a higher total recovery compared to their parallel experiment conducted at 60 °C, except for the nano flood in water-wet core (Test 2 compared to Test 7). However, in Test 2 crude oil C was used, as opposed to crude oil D, which was used for the other experiments. Test 4 and 5 were two of the experiments with the largest recovery during low rate flooding. However, they were also two of the cores with the highest permeability, which, in turn, could be a contributing factor to an increased oil recovery.

##### Tertiary Recovery

**Figure 7:**
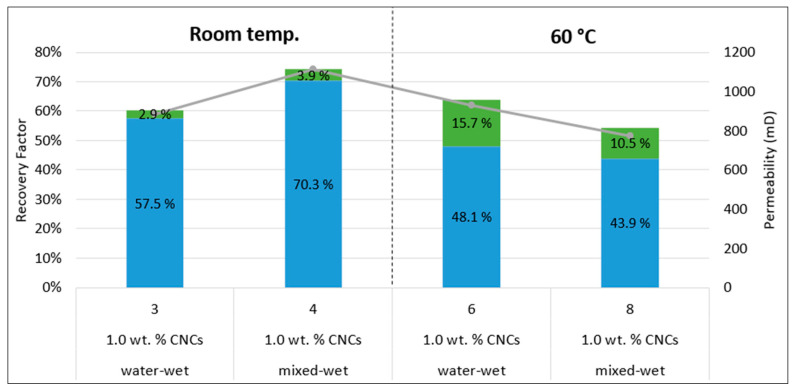
Recovery factors (% of OOIP) of tertiary recovery experiments. The vertical black dotted line acts as a marker between the experiments conducted at room temperature and 60 °C. Blue color represent the recovery factor achieved from low and high rate water flooding, while the green color denotes the recovery from the low and high rate nano flooding.

CNC nanofluid was introduced as a tertiary recovery technique, injected after water flooding, to see if incremental oil could be produced. With this injection mode, it would be more difficult for the nanoparticles to extract more oil, as most of the oil has already been recovered during water flooding. However, for all core floods, incremental oil was produced during the tertiary stage using CNC nanoparticles. The obtained results can be seen in Figure 7 and Table 10.

The four tests presented in Figure 7 are the same ones as already presented as secondary mode water floods in Figure 6. Thus, the focus of this part will be on the incremental oil production induced by the nanoparticles (green color in the figure).

In terms of oil recovery and wettability, the temperature had an effect as to which wettability conditions gave the highest incremental oil recovery. For the room temperature floods, the highest incremental recovery was observed for the mixed-wet rock. The opposite was seen when the temperature was increased, with the water-wet rock yielding the highest incremental oil recovery. However, as previously discussed in the secondary mode results, there is a permeability difference of 150 mD between Test 6 and Test 8, which could be an attributing factor to the difference in recovery. In addition, by looking at all permeability data (Figure 7), there seems to be a correlation between permeability and recovery, as recovery is decreasing when permeability is decreasing. Thus, more experiments should be conducted with cores, using different permeabilities to be able to confirm that the temperature plays a role regarding the wettability preference and oil recovery. Consequently, from these experiments, no trend can be concluded at this point, as permeability might be the dominating factor in the oil recovery, but not the wettability.

Regarding the effect of temperature, the largest increase in recovery was observed for the floods conducted at 60 °C, where 15.7% (Test 6) and 10.5% (Test 8) additional oil was produced. However, it should be noted that the room temperature floods had a much higher secondary mode (LSW flood) recovery compared to the LSW floods at 60 °C. Thus, more oil was available for the nanoparticles when implementing the tertiary stage at 60 °C. Nevertheless, by looking at the viscosity difference between LSW and 1.0 wt.% CNCs (Table 3), the difference is larger at 60 °C, which could be a contributing factor as to why more oil was produced at the high temperature.

To summarize the secondary and tertiary floods (Part 2), CNC nanoparticles were able to extract 2–27% of OOIP more oil than LSW when injected as a secondary technique, and they appeared to perform better under mixed-wet conditions. The oil recovery was enhanced when CNC nanofluid was injected as a tertiary recovery technique, where more incremental oil was produced for the high-temperature floods. For the tertiary floods, there did not seem to be an overall trend regarding rock wettability and oil recovery, as permeability might have been the dominating factor to the resulting high oil recoveries.

#### 4.4.3. Part 3

The effect of particle type was tested since CNCs have a different shape and size compared to T-CNFs (Figure 1). Thus, the two particles should result in a different behavior in the porous medium.

##### T-CNF and CNC Concentration

Two tertiary mode experiments were conducted, one using 0.1 wt.% T-CNFs (Test 10) and one using 1.0 wt.% CNCs (Test 3), both dispersed in 0.1 wt.% NaCl. The concentration of the two nanocellulose solutions differs from one another. A concentration of 0.1 wt.% T-CNFs was chosen, based on other flooding studies where T-CNFs have been employed [5].

CNCs were tested at different concentrations (0.5, 1.0, and 2.0 wt.%) in an injectivity study by Molnes et al. [7]. In their following work, they decided to move forward with the 0.5 wt.% CNCs, since it had the lowest differential pressure, but had still sufficiently effect on viscosity. The CNCs used in the current study are of the same quality as described by Molnes et al. [7]. Since 0.5 wt.% CNCs have already been documented in terms of EOR [6], it was of interest to test 1.0 wt.% CNCs, since it should give a higher viscosity effect, and might be able to recover more oil than the 0.5 wt.% CNCs. By looking at the tertiary mode experiments conducted in Part 2 in the current study, the CNC particles injected at 60 °C were attributable to an incremental oil recovery of 15.7% of OOIP and 10.5% of OOIP, respectively. No significant EOR effect was observed in the 60 °C experiment Molnes et al. [6] conducted using 0.5 wt.% CNCs.

A total of 0.1 wt.% CNCs were not considered a relevant concentration in the current experiments, as the effect of the particles was considered to be too low.

##### Oil Recovery Experiment

**Figure 8:**
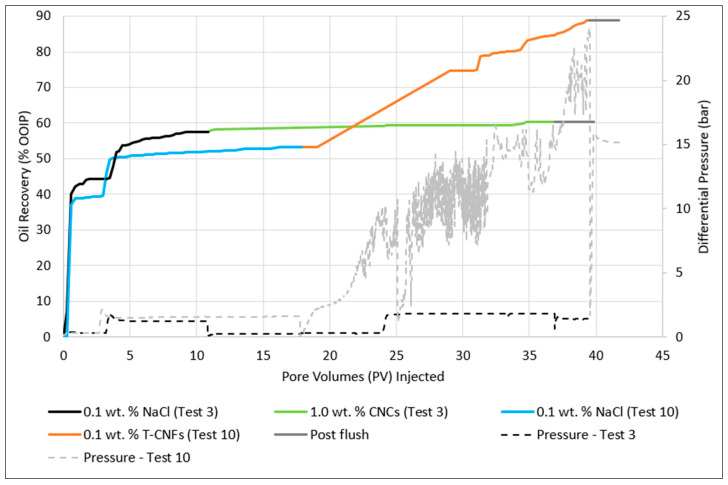
Recovery and corresponding pressure curves for 1.0 wt.% CNCs in 0.1 wt.% NaCl (Test 3) and 0.1 wt.% T-CNFs in 0.1 wt.% NaCl (Test 10).

From Table 10, it is seen that the core flood using CNCs gave a total recovery of 60.4%, where the nanofluid contributed to a 2.9% incremental oil recovery. For the core flood where T-CNFs were employed, an additional oil recovery of 35.4% of OOIP oil was observed during the tertiary stage, yielding a total recovery of 88.7% of OOIP (Table 10). Based on the calculated recovery, T-CNFs seems more promising for EOR applications. However, the differential pressure kept increasing throughout the T-CNF nano flood (Figure 8), even though no oil was produced towards the end of the flood. A similar pressure trend has also been observed in a study performed by Kusanagi et al. [5], using another quality of T-CNFs.

For the flooding with CNCs, the differential pressure was relatively stable at the end of both the low and high rate nano flood, where it was around 0.3 bar and 1.8 bar, respectively. For T-CNFs, there was a large response in pressure when the nanofluid injection started. The pressure quickly increased, but towards the end of the low rate, it stabilized around 12 bar. The differential pressure of the high rate injection continuously increased and finally reached a maximum pressure of 24.1 bar, which was approximately 11 times higher than what was observed for the high rate nano flood using CNC particles. At 24.1 bar it was decided to stop the T-CNF nano flood experiment, as the differential pressure was approaching the value of the surrounding sleeve pressure. 21 PVs of T-CNFs had been injected at that point. The fluctuation in pressure (spikes) together with the constant increase could indicate that log-jammings were building up and breaking free inside the porous medium, or that the fluid had created a filter cake on the inlet side. Log-jams are a form of mechanical entrapment of particles, which occurs when particles accumulate at pore throats leading to a blocked pore and reduced permeability, which in turn results in an increased differential pressure [3]. T-CNFs have a big aspect ratio, so the accumulation can be quite severe compared to CNCs. A filter cake was observed on the inlet side of the core after the experiment was done, which further supports the theory about particles being retained in the porous medium.

### 4.5. Microfluidics

**Figure 9:**
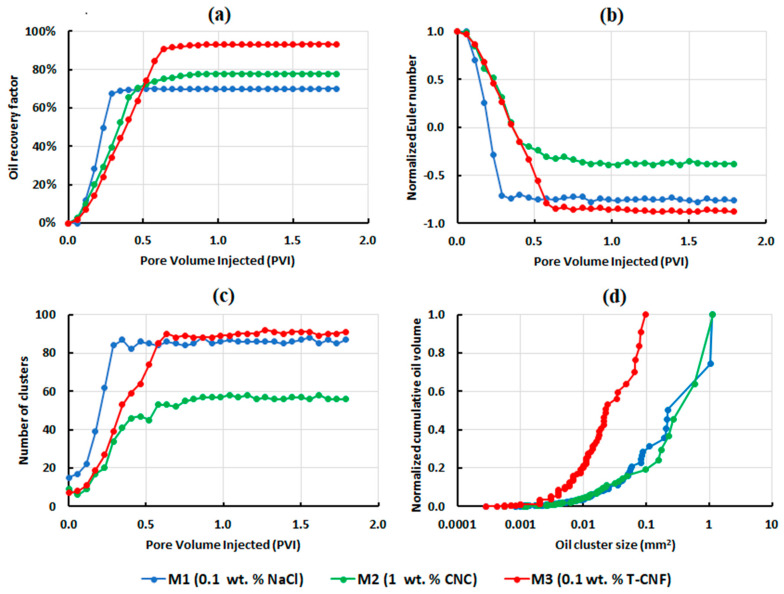
Results for the low rate injection microfluidic experiments: (**a**) oil recovery factor; (**b**) normalized Euler number; (**c**) number of clusters versus pore volume injected; and (**d**) normalized cumulative residual oil volume, as a function of oil cluster size.

The oil recovery factor was 71.7%, 77.7%, and 93.2% for test M1 (0.1 wt.% NaCl), M2 (1.0 wt.% CNCs) and M3 (0.1 wt.% T-CNFs), respectively, as shown in Figure 9a.

The highest and lowest connectivity of the residual oil was observed in the CNC flood (X = −0.38) and T-CNF flood (X = −0.87), respectively (Figure 9b). In experiment M1, the LSW flood resulted in a residual oil connectivity of −0.63. This flood had a faster disconnecting rate, which was due to the poor sweep efficiency compared to the other two floods, and consequently, the oil recovery started earlier for LSW.

**Figure 10:**
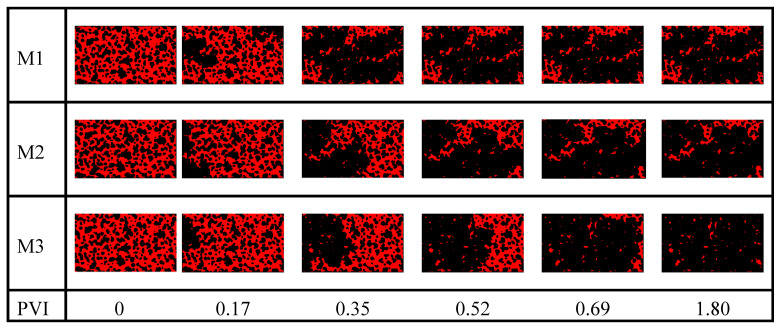
Change in the oil (in red) configuration in the analyzed part of the microchip as a function of pore volume injected (PVI) for experiment M1, M2, and M3.

T-CNFs significantly reduced the size of the remaining oil clusters compared to CNCs and LSW, by breaking up large oil clusters and then mobilizing them. This was evident from the number of clusters and size of the remaining clusters shown in Figure 9c,d. The LSW flood resulted in almost the same number of clusters as the T-CNF flood, but the system had a high trapping efficiency of the largest and smallest oil clusters. The CNC flood showed a higher reduction in oil saturation by 6% with less fraction of large remaining oil clusters compared to the LSW flood. It significantly attributed to a better sweep efficiency. Figure 10 shows the change in oil distribution in the analyzed part of the microchip as a function of pore volume injected (PVI) for experiments M1, M2, and M3, where it can also be seen that T-CNFs performed better than the two other fluids in improving the oil recovery.

**Figure 11:**
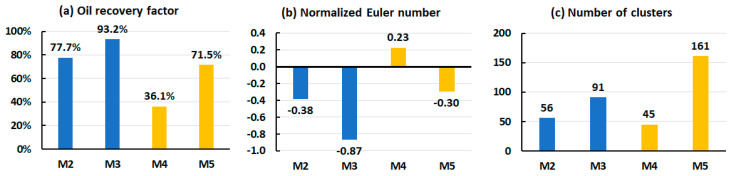
(**a**) Oil recovery, (**b**) normalized Euler number, and (**c**) number of clusters in the post-flooding images of low rate (in blue) and high rate (in yellow) microfluidic experiments. CNCs were used in M2 and M4, and T-CNFs were used in M3 and M5.

The effect of flow rate was tested by injecting CNCs (M4) and T-CNFs (M5). The applied flow rate was 10 times higher than the rate used in the corresponding parallel experiment, M2 (CNCs) and M3 (T-CNFs), respectively. A higher rate resulted in lower oil recovery factors and higher remaining oil connectivity as shown in Figure 11a,b. In addition, it was found that increasing the injection rate increased the residual number of clusters produced by T-CNFs, while the CNC flood led to a lower number of clusters, as illustrated in Figure 11c.

**Figure 12:**
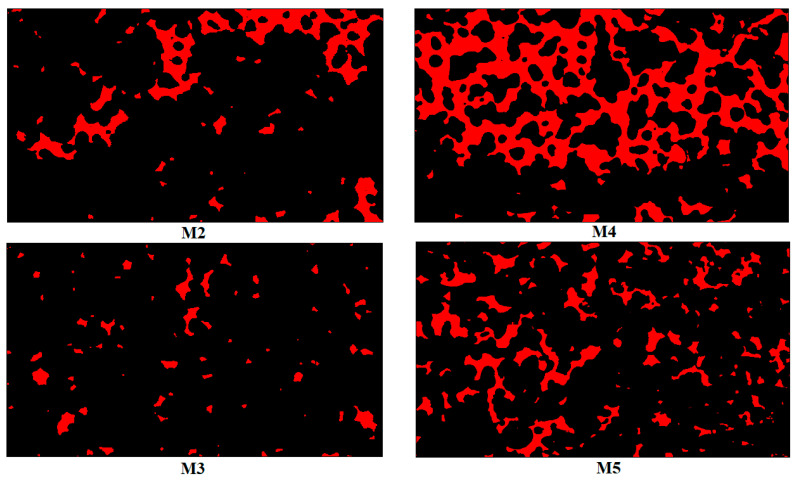
Illustration of the reimaging oil post flooding in CNCs experiments (M2, M4) and T-CNFs experiments (M3, M5). M4 and M5 were performed with 10 times higher injection rate, i.e., at 1.8 µL/min vs. 0.18 µL/min flow rate.

Figure 12 illustrates the post-flooding images corresponding to experiments M2–5, and it is clear that increasing the flow rate resulted in trapping of larger oil clusters, particularly for CNCs, where a very large cluster remained in the analyzed part of the microchip.

## 5. Conclusions

In this study, ten core floods and five microfluidic experiments were conducted to investigate the potential nanocelluloses have as a chemical EOR additive in low salinity water. Two types of nanocellulose particles were tested: cellulose nanocrystals and TEMPO-oxidized cellulose nanofibrils. In the study, the effect of injection scheme, temperature, wettability, and particle type were looked upon. In addition, interfacial tension and contact angle measurements were conducted to further support the findings from the floods. Based on the study the following conclusions can be drawn:

- The interfacial tension and contact angle values were dependent upon the crude oil type and nanoparticle type that was used. Overall, the IFT was not altered by the addition of T-CNF nanoparticles, but a small decrease in IFT was observed when CNCs were employed. For the contact angle, a slight increase in value was observed when CNCs or T-CNFs were added to the LSW. However, the change was marginal, thus, wettability alteration is not a primary EOR mechanism.
- For the secondary mode experiment where the core was re-used, the wettability restoration method was considered successful since it led to the same irreducible water saturation after primary drainage. From the oil recovery experiment, nanofluid yielded 5.8% of OOIP more oil, compared to low salinity water.
- CNC nanoparticles were able to extract 2–27% of OOIP more oil than LSW when injected as a secondary technique. Furthermore, the particles appeared to perform better under mixed-wet conditions.
- The oil recovery was enhanced when CNC nanofluid was injected as a tertiary recovery technique, where more incremental oil was produced for the high-temperature floods. For the tertiary floods, there did not seem to be an overall trend regarding rock wettability and oil recovery.
- Looking at the effect of particle type, T-CNFs were much more effective than CNCs to recover trapped oil. This was evident from both core flooding and microfluidic experiments. However, the pressure was constantly increasing during the high rate T-CNF core flood. Even though more oil was recovered during the T-CNF flood, the high pressure indicates poor injectivity. Furthermore, filtering of particles was observed on the inlet side of the core plug after the experiment. Future experiments should, therefore, test T-CNFs at a lower concentration, to see if a similar high incremental oil recovery can be achieved with a lower and more stable pressure profile.
- The microfluidic experiments supported the findings from the core floods, with nanofluid leading to a better sweep efficiency compared to low salinity flooding. T-CNFs improved the oil recovery the most, by breaking up large oil clusters and mobilizing them. Looking at the effect of the flow rate, it was evident that a higher flow rate resulted in lower oil recovery factors and higher remaining oil connectivity.

## Figures and Tables

**Table 1:** Properties of the cores used in experiments.

Core	Length	Diameter	Pore Volume	Permeability	Porosity
(no.)	(cm)	(cm)	(mL)	(mD)	(%)
1	10.0	3.8	19.3	781	17.5
2	10.0	3.7	18.3	896	15.1
3	9.9	3.8	17.5	883	15.9
4	9.7	3.8	18.4	1111	17.1
5	9.9	3.8	18.5	1067	16.9
6	9.9	3.8	18.8	928	17.2
7	9.9	3.8	18.8	768	17.2
8	9.9	3.8	18.1	771	16.5
9	9.9	3.8	18.3	727	16.8
10	9.9	3.8	17.5	832	16.0

**Table 2:** Fluid properties.

Fluid	Density (g/cm^3^)		Viscosity (cP)	
	24 °C	60 °C	24 °C	60 °C
0.1 wt.% NaCl	1.00	0.98	0.91	0.47
1 wt.% CNCs in 0.1 wt.% NaCl	1.00	1.01	1.40	1.09
0.5 wt.% CNCs in 0.1 wt.% NaCl	0.99	1.00	1.33	1.24
0.1 wt.% T-CNFs in 0.1 wt.% NaCl	1.01	-	3.67	-
Crude oil C	0.91	0.89	55.90	12.19
Crude oil D	0.89	0.87	20.74	5.88

**Table 3:** Charge density and properties of the nanocellulose qualities.

Sample	Charge Density * (mmol/g)	Functional Groups in Significant Amounts	Zeta Potential	Apparent Size by DLS (nm)
CNCs	approx. 0.3 **	–OH, –SO_3_H	−40.1 ± 2.5	123 ± 0–164 ± 2 nm
T-CNFs	1.13	–OH, –COOH, –CHO	−41.7 ± 2.2	1019 ± 297 nm

* This is carboxylic acids for T-CNFs and sulphate half ester for CNCs. ** Determined by ICP-AA. The procedure for zeta potential and apparent size by dynamic light scattering (DLS) can be found in Aadland [54].

**Table 4:** Saturates, aromatics, resins, asphaltenes (SARA) analysis of crude oil.

Type of Oil	Weight Percent (Normalized)			
	Saturates	Aromatics	Resins	Asphaltenes
Crude oil C	66.21	25.78	7.69	0.32
Crude oil D	71.57	20.81	7.44	0.18

**Table 5:** Overview of the different tests conducted. The letters A and B denote that the experiments were conducted on the same core plug. The test number corresponds to the core number in Table 1.

Part	Test No.	Fluids			Conditions	
		Secondary Agent	Tertiary Agent	Oleic Phase	Temp. (°C)	Aging Time (Weeks)
1	1A	0.1 wt.% NaCl	-	Crude oil C	24	-
	1B	1.0 wt.% CNCs	-			-
2	2	1.0 wt.% CNCs	-			-
	3	0.1 wt.% NaCl	1.0 wt.% CNCs	Crude oil D		-
	4	0.1 wt.% NaCl	1.0 wt.% CNCs			5
	5	1.0 wt.% CNCs	-			5
	6	0.1 wt.% NaCl	1.0 wt.% CNCs		60	-
	7	1.0 wt.% CNCs	-			-
	8	0.1 wt.% NaCl	1.0 wt.% CNCs			5
	9	1.0 wt.% CNCs	-			7
3	10	0.1 wt.% NaCl	0.1 wt.% T-CNF		24	-

**Table 6:** Details of injection and image acquisition for microfluidics experiments.

Test No.	Recovery Agent	Flow Rate (µL/min)	Capillary Number	Duration (min)	Pore Volume Injected	Analyzed Area (mm^2^)	Image Number
M1	0.1 wt.% NaCl	0.18	1.2 × 10^−6^	23.0	1.8	28.1	31
M2	1.0 wt.% CNCs		2.1 × 10^−6^				
M3	0.1 wt.% T-CNFs		5.0 × 10^−6^				
M4	1.0 wt.% CNCs	1.80	2.1 × 10^−5^	10.2	8.0		25
M5	0.1 wt.% T-CNFs		5.0 × 10^−5^				

**Table 7:** Interfacial tension (IFT) and contact angle values. Each experiment lasted for 12 h. The average value from the last hour of the experiment is reported in the table. Measurements were made at ambient conditions.

Fluid	Interfacial Tension (mN/m)		Contact Angle (°)	
	Crude Oil C	Crude Oil D	Crude Oil C	Crude Oil D
0.1 wt.% NaCl	19.2 ± 0.02	15.3 ± 0.03	49.6 ± 0.2	52.5 ± 0.8
1.0 wt.% CNCs	16.9 ± 0.02	13.8 ± 0.05	52.0 ± 0.1	56.1 ± 0.2
0.1 wt.% T-CNFs	19.1 ± 0.07	15.4 ± 0.03	51.1 ± 0.1	60.3 ± 0.1

**Table 8:** Overview of capillary numbers for core floods. * The reported capillary number is an average based on all the floods conducted with the respective fluid. For these floods, the capillary numbers were all in the same order of magnitude.

Fluid	Capillary Number for Core Floods			
	Crude Oil C		Crude Oil D	
	0.3 mL/min	3.0 mL/min	0.3 mL/min	3.0 mL/min
0.1 wt.% NaCl	1.23 × 10^−6^	1.23 × 10^−5^	1.6 × 10^−6^ *	1.6 × 10^−5^ *
1.0 wt.% CNCs	2.50 × 10^−6^	2.50 × 10^−5^	2.7 × 10^−6^ *	2.7 × 10^−5^ *
0.1 wt.% T-CNFs	-	-	6.0 × 10^−6^	6.0 × 10^−5^

**Table 9:** Pore volume of core 1 after cleaning, together with permeability and porosity data before and after water flood and core cleaning.

Core	New Pore Volume	Porosity (%)		Permeability (mD)		Reduction Perm.
	(mL)	Before	After	Before	After	(%)
1	19.2	17.5	17.3	781	731	6.5

**Table 10:** Summary of initial water saturations (S_wi_) and recovery factors (as% OOIP) for the core floods for each of the flooding stages (low rate (Q_low_), 0.3 mL/min and high rate (Q_high_), 3.0 mL/min). * This test has been used for comparison with an experiment in Part 3.

Part	Test No.	Fluids			Conditions			Incremental Recovery Factor of OOIP (%)				Total Recovery (%)
		Secondary Agent	Tertiary Agent	Crude Oil Type	T	Aging Time	Swi	Secondary Agent		Tertiary Agent		
					(°C)	(Weeks)	(Fraction)	Q_low_	Q_high_	Q_low_	Q_high_	
1	1A	0.1 wt.% NaCl	-	C	24	-	0.247	40.9	11.1	-	-	52.0
	1B	1.0 wt.% CNCs	-			-	0.241	48.6	9.2	-	-	57.8
2	2	1.0 wt.% CNCs	-			-	0.314	48.6	10.5	-	-	59.0
	3 *	0.1 wt.% NaCl	1.0 wt.% CNCs	D		-	0.306	44.3	13.2	1.7	1.2	60.4
	4	0.1 wt.% NaCl	1.0 wt.% CNCs			5	0.308	66.8	3.5	3.6	0.3	74.2
	5	1.0 wt.% CNCs	-			5	0.302	80.2	1.0	-	-	81.2
	6	0.1 wt.% NaCl	1.0 wt.% CNCs		60	-	0.407	42.8	5.3	9.4	6.3	63.8
	7	1.0 wt.% CNCs	-			-	0.383	54.8	9.4	-	-	64.2
	8	0.1 wt.% NaCl	1.0 wt.% CNCs			5	0.173	39.8	4.0	2.7	7.8	54.4
	9	1.0 wt.% CNCs	-			7	0.311	63.9	7.2	-	-	71.1
3	10	0.1 wt.% NaCl	0.1 wt.% T-CNFs		24	-	0.411	39.4	14.0	25.5	9.9	88.7
	3	0.1 wt.% NaCl	1.0 wt.% CNCs			-	0.306	44.3	13.2	1.7	1.2	60.4
